# Seasonal adaptive strategies of North Tinerfeña goats in the Canary Islands, Spain

**DOI:** 10.1007/s00484-026-03313-2

**Published:** 2026-10-06

**Authors:** Wallace Sostene Tavares da Silva, Robson Mateus Freitas Silveira, Luis Alberto Bermejo, Alexandr Torres Krupij, Débora Andréa Evangelista Façanha

**Affiliations:** 1 Department of Animal Science, Federal Rural University of the Semi-Arid Region (UFERSA), Mossoró, RN 59625-900 Brazil; 2 Department of Animal Science, “Luiz de Queiroz” Agriculture College (ESALQ), University of São Paulo (USP), Piracicaba, São Paulo State 13418-900 Brazil; 3 Departamento de Ingeniería Agraria y del Medio Natural, Escuela Politécnica Superior de Ingeniería – Sección Agraria, Universidad de La Laguna (ULL), Carretera de Geneto nº2, San Cristóbal de La Laguna (Tenerife), 38200 España; 4 Animal Production Unit, Pastures and Forages in Arid and Subtropical Zones, Canarian Institute of Agricultural Research (ICIA), PBOX n° 60, La Laguna, Santa Cruz de Tenerife, 38200 España; 5 University of International Integration of Afro-Brazilian Lusophony (UNILAB), Redenção, CE 62790-790 Brazil

**Keywords:** Goat adaptation, Thermoregulation, Coat traits, Heat stress, Local breeds

## Abstract

Climate change has intensified thermal challenges in livestock production systems, reinforcing the need to understand adaptive mechanisms in locally adapted breeds. This study aimed to characterize the seasonal adaptive profile of North Tinerfeña goats, an autochthonous breed from the Canary Islands. Thirty adult non-pregnant female goats were evaluated under standardized management conditions. Repeated measurements of rectal temperature (RT), respiratory rate (RR), and coat surface temperature (CST) were obtained twice daily, in the morning and afternoon, throughout a one-year study period encompassing winter and summer. Coat characteristics (coat thickness [CT], hair length [HL], hair diameter [HD], and hair angle) were measured once per day during winter and summer. Physiological variables were significantly affected by season and period of the day (*p* < 0.001), with a significant season × period interaction for RR and CST (*p* < 0.01). Coat traits showed pronounced seasonal variation, with CT significantly greater in winter (*p* < 0.001) and HL significantly greater in summer (*p* < 0.001), whereas HD did not differ between seasons (*p* > 0.05). Multivariate analyses revealed distinct seasonal adaptive patterns, with CT and HL identified as the main discriminant variables between winter and summer (discriminant analysis, *p* < 0.001). Decision tree (CHAID) analysis identified coat thickness as the primary classifier of seasonal condition, with a threshold value of CT ≤ 8.7 mm predominantly characterizing summer (*p* < 0.001). These findings demonstrate that North Tinerfeña goats rely on integrated and season-dependent thermoregulatory strategies, in which morphological coat traits predominate during winter, while rapid and sensitive physiological responses are preferentially activated during summer to maintain homeothermy. This study provides novel phenotypic evidence of local adaptation and highlights the potential use of objective morphological thresholds, such as coat thickness, combined with simple physiological indicators, to support management and conservation strategies for insular goat breeds under climate change scenarios.

**Supplementary Information:**

The online version contains supplementary material available at https://doi.org/10.1007/s00484-026-03313-2.

## Introduction

Global climate change has intensified the occurrence of extreme thermal events, increasing the frequency of heat waves and seasonal temperature variability, with direct impacts on animal welfare, productivity, and the sustainability of livestock production systems. Small ruminants, particularly goats, are often considered resilient species; however, this resilience largely depends on breed-specific morphological, physiological, and behavioral adaptations shaped by the environments in which they evolved (Silveira et al. 2026). Recent reviews emphasize that understanding these adaptive mechanisms is essential to mitigate the effects of heat stress under climate change scenarios (Berihulay et al. 2019; Joy et al. 2020; Gaughan et al. 2018).

Thermal adaptation in goats results from the interaction among anatomical traits, behavioral adjustments, and physiological responses (Sejian et al. 2018). Coat characteristics—such as thickness, length, diameter, and hair angle—directly influence solar radiation absorption and sensible and latent heat exchange (Ribeiro et al. 2016; Costa et al. 2018). Concurrently, physiological responses including increased respiratory rate, peripheral vasodilation, and changes in coat temperature enable heat dissipation when morphological limits are exceeded (Lima et al. 2022). The literature highlights that these mechanisms act in an integrated manner and that their relative contribution may vary seasonally and among breeds (Nair et al. 2021; Joy et al. 2020).

Local and insular breeds have received increasing attention due to their potential role in adapting livestock production to climate change (Silveira et al. 2026). These populations often exhibit specific phenotypic adaptations, shaped by long periods of natural selection under restrictive environmental conditions such as limited water availability, rugged terrain, and high thermal variability (Façanha et al. 2023; da Silva et al. 2025a, b). Despite their importance, many autochthonous breeds remain poorly characterized from a functional perspective, limiting effective management, conservation, and valorization strategies for these genetic resources.

The North Tinerfeña goat is an autochthonous breed from the Canary Islands, traditionally raised under extensive and semi-extensive systems, in environments characterized by marked seasonal thermal variation, intense solar radiation, and restricted forage availability. Genetic studies on Canary Island goats indicate the presence of significant genetic diversity and signals of local adaptation, reinforcing their value as zoogenetic resources (Senczuk et al. 2024).

Although previous studies have described physiological responses and coat characteristics associated with thermal adaptation in goats (da Silva et al. 2025a, b), the seasonal adaptive mechanisms of the North Tinerfeña breed remain largely unknown. In particular, there is a lack of information on how morphological and physiological thermoregulatory mechanisms interact across contrasting seasons to maintain thermal balance in this insular population. Therefore, the novelty of the present study lies in providing the first integrated characterization of seasonal adaptive responses in North Tinerfeña goats by simultaneously evaluating environmental conditions, coat traits, and physiological indicators. This approach advances current knowledge by identifying seasonal shifts in thermoregulatory strategies and by generating functional evidence of local adaptation in a breed for which such information is currently unavailable.

It is hypothesized that North Tinerfeña goats exhibit a distinct seasonal adaptive profile, in which morphological mechanisms associated with coat traits predominate during colder periods, promoting heat conservation, whereas sensitive and rapid physiological mechanisms are preferentially activated during warmer periods, facilitating heat dissipation and the maintenance of homeothermy.

The objective of this study was to characterize the seasonal adaptive profile of the North Tinerfeña breed, integrating anatomical coat traits, thermoregulatory physiological responses, and environmental variables, in order to contribute to the understanding of thermal adaptation mechanisms in this breed and to provide support for management and conservation strategies under climate change scenarios.

## Materials and methods

### Study area and period

The study was conducted at the Canary Islands Institute of Agrarian Research (ICIA), an autonomous body of the Government of the Canary Islands, linked to the Ministry of Agriculture, Livestock, Fisheries and Food, located on Tenerife Island, Canary Islands, Spain.

Tenerife is situated at 28°19′ N and 16°34′ W and is the largest and highest island of the Canary Archipelago. The island has predominantly volcanic soils and a Mediterranean climate with subtropical influence, characterized by low annual temperature variability (approximately 18–26 °C) and low annual precipitation (about 400–1000 mm). Climatic conditions are strongly influenced by northeasterly trade winds associated with the Azores anticyclone, which moderate temperatures throughout the year.

Vegetation on the island is highly heterogeneous due to pronounced altitudinal gradients, ranging from succulent-dominated communities at low altitudes to dry zones, lowland forests, and high-altitude vegetation above 1500 m.

## Animals

Data were collected from 30 North Tinerfeña goats, all females, aged between 2 and 4 years and non-pregnant, with an average body weight of 39.45 ± 3.58 kg. The goats were maintained under an intensive production system. Animals were housed in facilities roofed with metal sheets, laterally open on the longest side (4 m), with concrete flooring. Goats were grouped in elevated stalls equipped with metal feeders, and water was provided ad libitum.

The animals were managed according to the routine sanitary practices adopted on the farm. Preventive health care included regular vaccination and deworming programs, as well as periodic clinical monitoring to ensure animal health throughout the experimental period. No clinical signs of disease were observed during the study.

Concentrate feed based on corn and soybean meal was supplied during the early morning hours (06:00 h), and chopped forage composed of native plant species was offered throughout the day. Diets met the nutritional requirements recommended for small ruminants.

## Data collection

Repeated measurements were conducted on the same animals and at the same experimental site, allowing the assessment of thermoregulatory and environmental responses under standardized conditions. The study was conducted over a 12-month period in the Canary Islands, Spain, encompassing both winter and summer. Physiological measurements were obtained twice daily, at 08:00 h and 14:00 h, throughout the experimental period. Prior to each data collection session, animals were kept in a single holding pen for 30 min to minimize the influence of handling and acclimation stress on physiological responses. Measurements were obtained in the following order: respiratory rate, rectal temperature, coat surface temperature, and environmental variables, which were recorded simultaneously, followed by the assessment of morphological traits.

## Thermoregulatory responses

Respiratory rate (RR) was measured by stethoscope auscultation over 1 min. Rectal temperature (RT) was obtained with a digital clinical thermometer (up to 44 °C), inserted ~ 5 cm into the rectum, in contact with the mucosa. Coat surface temperature was measured using a handheld infrared thermometer positioned approximately 10 cm from the animal’s coat surface. Measurements were obtained from the lateral thoracic region and recorded immediately for subsequent analysis.

## Coat morphological traits

Coat samples were collected from the flank region using duckbill pliers to determine hair length and hair diameter. Coat thickness was measured in situ at the same anatomical site using a millimetric metal ruler equipped with a slider, inserted perpendicularly through the coat until contacting the skin surface. The slider was then adjusted to the outermost layer of the coat, and the distance was recorded.

Hair length was determined in the laboratory using a digital caliper. Following the methodology proposed by Udo (1978), the 10 longest hairs within each sample were measured, and their arithmetic mean was used to represent coat length. This procedure is widely employed in studies of animal coat characteristics because the longest fibers are considered the main contributors to the thermal insulation properties of the coat. Hair diameter was measured on the same fibers using a digital micrometer, and the arithmetic mean was subsequently calculated (Lee 1953).

Hair angle (rad) was measured in situ in the flank region using a metal protractor, following the methodology described by Silva (2008). The variable was expressed in radians and represents the angle formed between the hair shaft and the skin surface. Biologically, greater hair angles indicate a more erect coat structure, which may increase the insulating air layer retained within the coat, whereas lower angles indicate hairs positioned closer to the skin surface, potentially facilitating heat dissipation.

### Environmental variables

Air temperature and relative humidity were recorded using a digital thermo-hygrometer and black globe thermometer in the same setting and time as the morphophysiological measurements, yielding paired ambient data per animal. From these readings, the Radiant Heat Load (RHL) was calculated according to Silva (2008):

$$RHL=1.53\times hc\times\left(TGN-Ta\right)+\sigma TGN^4\left(W/m^2\right)$$

where: hc = convective heat transfer coefficient (W/m²·K); TGN = black globe temperature (K); Ta = air temperature (K); σ = Stefan–Boltzmann constant (5.6697 × 10⁻⁸ W/m²·K⁴).

Environmental data and the calculated radiant heat load are provided in Table 1.

**Table 1:** Mean, standard error of the mean (SEM), minimum, and maximum values of air temperature (AT), relative humidity (RH), and radiant heat load (RHL) recorded during winter and summer in the Canary Islands, Spain.

		Mean	SEM	Minimum	Maximum
AT, °C	Winter	22.80	0.38	16.30	32.22
	Summer	28.71	0.44	21.60	39.80
RH, %	Winter	56.61	1.19	28.00	82.00
	Summer	49.34	1.07	25.00	70.00
W m⁻²	Winter	420.59	2.21	388.00	504.21
	Summer	456.74	2.26	424.71	566.79

## Statistical methods

Data were initially evaluated for normality of residuals and homogeneity of variances using the Shapiro–Wilk and Levene tests, respectively. The sample size was determined based on an a priori statistical power analysis, which indicated that the number of animals included in the study was sufficient to detect biologically relevant differences with adequate statistical power. Furthermore, all assumptions required for the applied statistical procedures were verified and met prior to analysis. Repeated measurements obtained from the same animals across seasons and periods of the day were appropriately considered in the statistical models used to evaluate physiological responses. For multivariate analyses, including exploratory factor analysis (EFA), discriminant analysis, and CHAID, data suitability was confirmed through the respective diagnostic procedures, ensuring the robustness of the identified patterns. Although the evaluated sample represents a relatively limited population, it is consistent with studies involving local breeds and repeated-measures designs (Silveira et al. 2024; da Silva et al. 2025a, b).

Physiological variables—rectal temperature (RT), respiratory rate (RR), and coat surface temperature (CST)—were analyzed using a repeated-measures analysis of variance (ANOVA) in a factorial model, considering the fixed effects of season (winter and summer), period of the day (morning and afternoon), and the season × period interaction. When significant differences were detected, means were compared using Tukey’s test, adopting a significance level of 5% (*p* ≤ 0.05).

Coat characteristics—coat thickness (CT), hair length (HL), hair diameter (HD), and hair angle—were compared between seasons using a repeated-measures one-way ANOVA, considering season as a fixed effect. Means were presented with their respective standard deviations, and statistical differences were considered significant when *p* ≤ 0.05.

For the multivariate analyses, repeated observations obtained from each animal within each season were averaged to avoid pseudoreplication, with the animal × season combination defined as the analytical unit. This aggregation resulted in 60 animal-season records, representing 30 goats evaluated during both winter and summer.

To investigate multivariate relationships among physiological, environmental (AT, RH, and RHL), and coat variables, exploratory factor analysis was performed separately for winter and summer. Only components with eigenvalues greater than 1 were considered relevant. The Kaiser–Meyer–Olkin (KMO) measure and Bartlett’s test of sphericity were verified and validated. Varimax rotation was applied.

Linear discriminant analysis was performed to assess multivariate separation between seasons and identify the physiological and morphological traits that contributed most strongly to seasonal differentiation. Season, classified as winter or summer, was used as the grouping variable. Because only two groups were compared, a single canonical discriminant function was estimated. The function was characterized by its eigenvalue, canonical correlation, Wilks’ lambda, and group centroids. The relative contribution of each predictor was evaluated using standardized canonical coefficients and structure loadings. Predictive performance was assessed using grouped leave-one-animal-out cross-validation. In each iteration, both seasonal records from the same animal were simultaneously excluded from the training dataset and used for validation, ensuring that records from the same goat were not shared between the training and validation sets. Predictor standardization and model fitting were performed independently within each training iteration. Classification performance was summarized using the confusion matrix, overall accuracy, and the percentage of correctly classified records for each season.

A decision tree (CHAID) was applied to classify animals according to season, considering physiological and coat variables. Split criteria were based on the chi-square (χ²) test, with adjusted *p*-values and a significance level of 5%.

## Results

### Physiological parameters as a function of period of the day and season

Rectal temperature (RT) was influenced by both period of the day and season (*p* < 0.001 for both), with no significant period × season interaction (*p* = 0.54) (Table 2). Regardless of season, higher RT values were observed in the afternoon (39.25 °C) compared with the morning (38.99 °C). Considering seasonal effects, mean RT was higher in summer (39.16 °C) than in winter (39.07 °C) (*p* < 0.001). Respiratory rate (RR) was significantly affected by period, season, and their interaction (*p* < 0.001). During summer, RR was substantially higher, particularly in the afternoon (54.13 breaths min⁻¹), whereas in winter lower values were observed both in the morning (30.71 breaths min⁻¹) and afternoon (33.83 breaths min⁻¹). Coat surface temperature (CST) was also influenced by period of the day and season (*p* < 0.001), with a significant interaction (*p* = 0.002). The highest CST values were recorded during summer, especially in the afternoon (32.79 °C), whereas the lowest values occurred in winter during the morning (26.19 °C).

**Table 2:** Mean values of rectal temperature (RT, °C), respiratory rate (RR, mov. min⁻¹), and coat surface temperature (CST, °C) of North Tinerfeña goats according to season (winter and summer) and period of the day (morning and afternoon) (n= 30 goats)

Variables	Periods	Seasons			SEM	p-value		
		Winter	Summer	Mean		Period (P)	Season (S)	P*S
RT	Morning	39.16	38.81	38.99b	0.03	< 0.001	< 0.001	0.54
	Afternoon	38.99	39.51	39.25a				
	Mean	39.07b	39.16a					
RR	Morning	30.71bB	38.64aB	34.68	0.79	< 0.001	< 0.001	< 0.001
	Afternoon	33.83bA	54.13aA	43.98				
	Mean	32.27	46.39					
CST	Morning	26.19bB	28.59aB	27.39	0.17	< 0.001	< 0.001	0.002
	Afternoon	28.94bA	32.79aA	30.87				
	Mean	27.56	30.69					

### Coat characteristics as a function of season

Coat thickness (CT) was significantly greater in winter (11.68 mm) compared with summer (1.99 mm) (*p* < 0.001; Table 3). Similarly, hair length (HL) showed higher values in summer (146.69 mm) than in winter (108.57 mm) (*p* < 0.001). Hair diameter (HD) did not differ between seasons (*p* = 0.590), maintaining similar mean values in winter and summer (0.074 mm). In contrast, hair angle differed significantly between seasons (*p* < 0.001), with higher mean values in winter (0.00075) compared with summer (0.00063).

**Table 3:** Coat characteristics of North Tinerfeña goats in winter and summer: coat thickness (CT), hair length (HL), hair diameter (HD), and hair angle (rad) (*n* = 30 goats)

Variables		Mean	SD	*P*-value
CT	Winter	11.68ª	2.51	< 0.001
	Summer	1.99^b^	3.02	
HL	Winter	108.57^b^	34.78	<0.001
	Summer	146.69ª	48.72	
HD	Winter	0.074ª	0.011	0.590
	Summer	0.074ª	0.008	
Angle	Winter	0.00075ª	0.00027	< 0.001
	Summer	0.00063^b^	0.00043	

^a, b^ Means within the same variable followed by different superscript letters differ significantly between winter and summer according to Tukey’s test (*P* < 0.05)

### Multivariate analysis of environmental, physiological, and coat variables

**Fig. 1:**
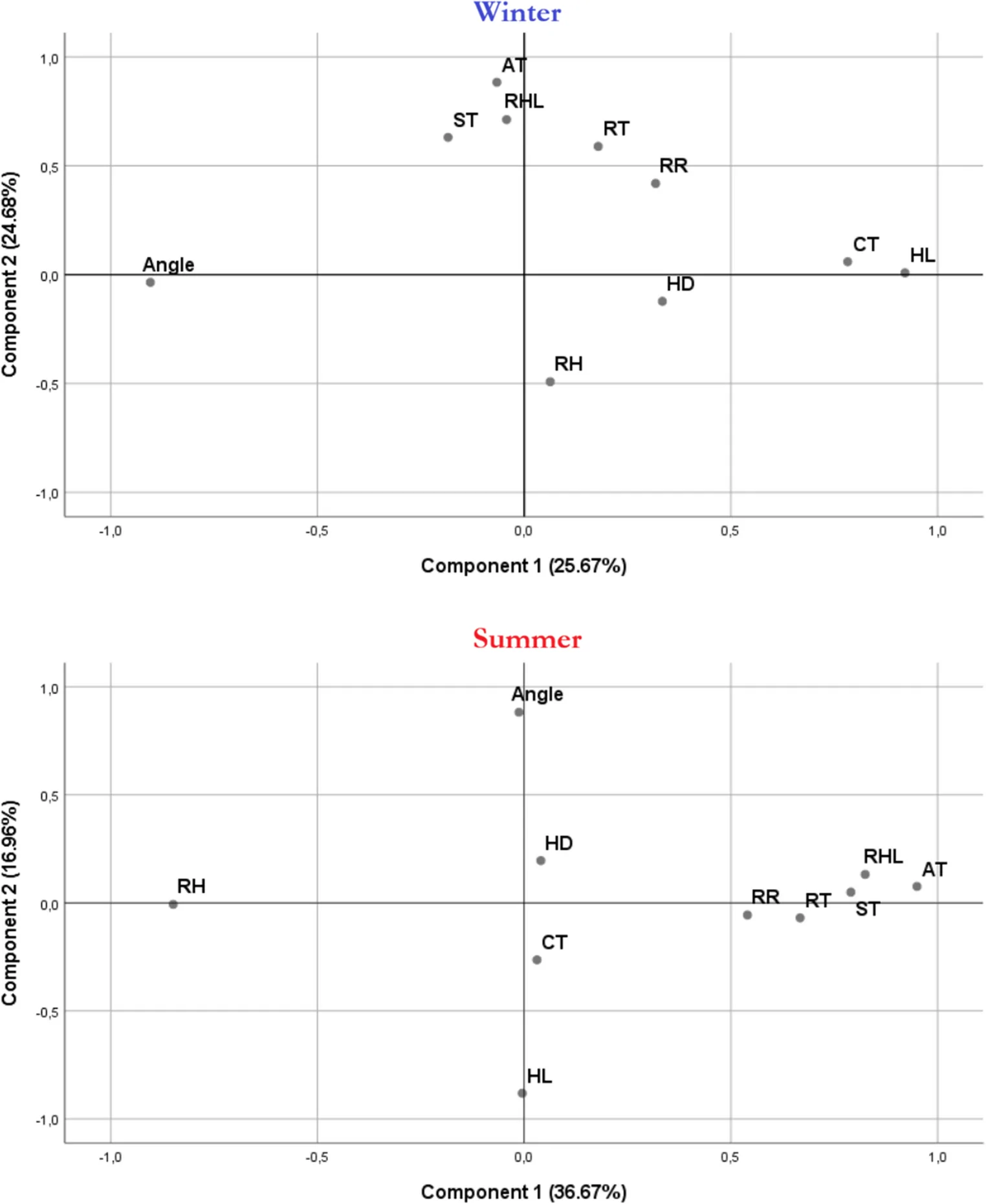
Factor analysis of physiological responses, environmental conditions, and coat traits in North Tinerfeña goats during winter and summer in the Canary Islands, Spain

 Exploratory Factor Analysis revealed distinct patterns between seasons. In winter, the first two components explained 50.35% of the total variance (Component 1: 25.67%; Component 2: 24.68%; Fig. 1), with stronger associations among RT, RR, and environmental variables, while CT and HL were positioned on the first component, indicating that morphological traits are the primary adaptive mechanisms used by North Tinerfeña goats for thermal adaptation during this season. In summer, the first two components explained 53.63% of the variance (Component 1: 36.67%; Component 2: 16.96%). A strong association among RT, RR, AT, CST, and RHL was observed on the first component, whereas CT and HL showed distinct loading patterns, highlighting their structural role in thermal insulation and modulation of environmental heat load.

The linear discriminant analysis revealed a clear multivariate separation between winter and summer (Supplementary Table). The single discriminant function had an eigenvalue of 12.059, a canonical correlation of 0.961, and a Wilks’ lambda of 0.077 (P < 0.001). The group centroids were −3.414 for winter and 3.414 for summer, indicating a marked separation between the two seasons along the discriminant function. Hair length and coat thickness showed the largest absolute standardized canonical coefficients (0.865 and 0.850, respectively), indicating that these variables made the greatest independent contributions to seasonal discrimination. Grouped leave-one-animal-out cross-validation correctly classified 59 of the 60 animal-season records, resulting in an overall accuracy of 98.3%. All summer records were correctly classified (30/30; 100.0%), whereas 29 of the 30 winter records were correctly assigned (96.7%). Only one winter record was misclassified as summer, demonstrating the high discriminatory performance of the model.

### Seasonal classification using decision tree analysis

**Fig. 2:**
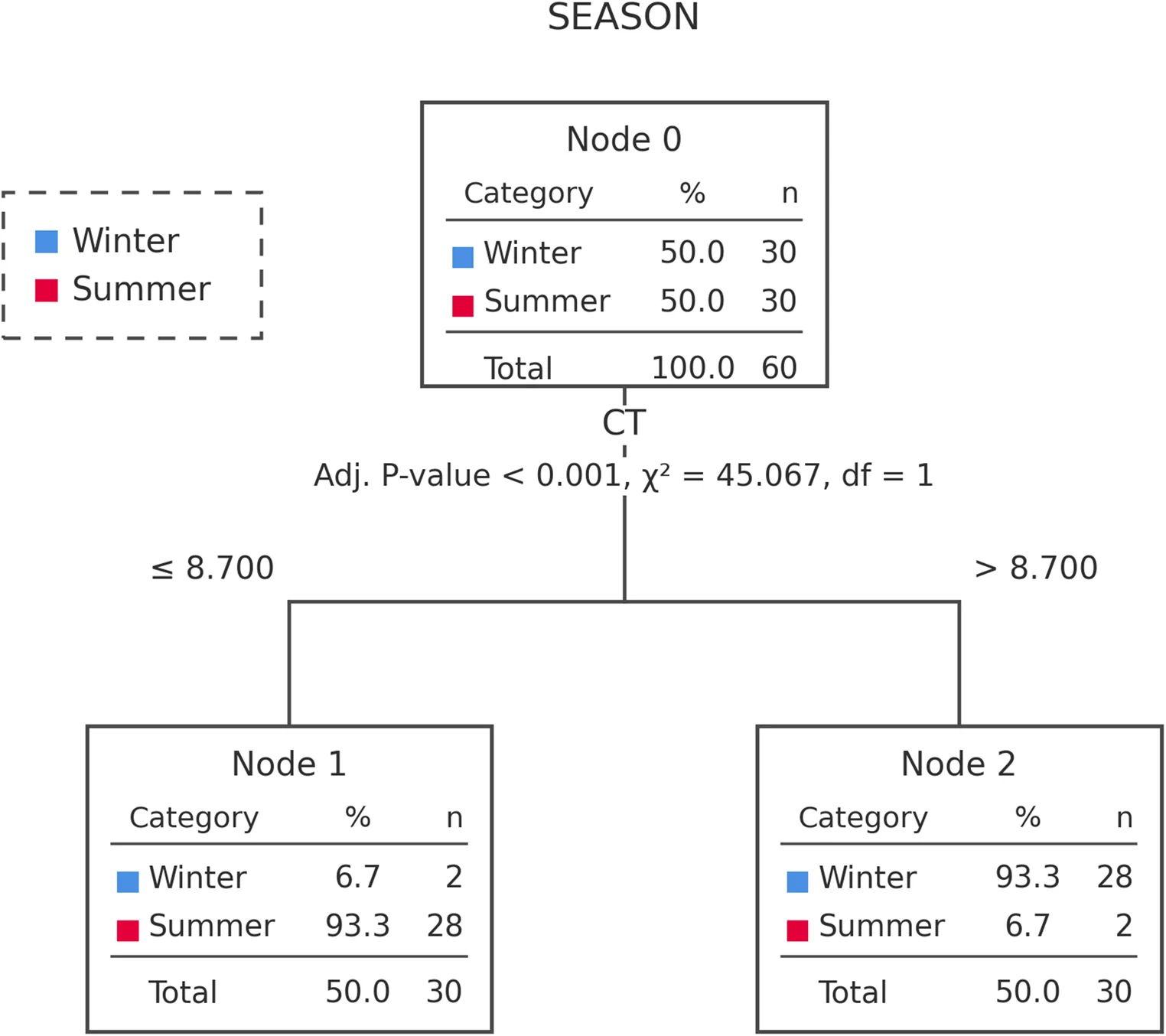
Decision tree (CHAID) for seasonal classification of North Tinerfeña goats

The CHAID decision tree revealed coat thickness (CT) as the most important variable for distinguishing seasons (*p* < 0.001; χ² = 45.067) (Fig. 2). The optimal split was observed at CT = 8.70. Values ≤ 8.70 were predominantly associated with summer, with 28 of 30 observations (93.3%) classified as summer. Conversely, CT values > 8.70 were predominantly associated with winter, with 28 of 30 observations (93.3%) classified as winter. These findings indicate a marked seasonal adjustment in coat thickness, characterized by a thinner coat during summer and a thicker coat during winter. 

## Discussion

This study describes, for the first time, the seasonal adaptive profile of North Tinerfeña goats in the Canary Islands. The findings indicate the existence of a seasonal adaptive pattern characterized by the use of different thermoregulatory strategies to maintain homeothermy. During winter, animals appear to prioritize morphological mechanisms, particularly coat-related traits, which contribute to heat conservation. In contrast, during summer, there is greater reliance on latent and sensitive physiological mechanisms to dissipate heat, reflecting a dynamic adaptive strategy in response to seasonal environmental variation.

The integration of coat and physiological responses also indicates that thermal adaptation in this breed cannot be explained by a single trait. Instead, it reflects the coordinated action of multiple phenotypic mechanisms that optimize heat conservation or dissipation according to environmental demands. Such adaptive flexibility is particularly relevant for local breeds, which have been subjected to long-term natural and human selection under specific environmental conditions. The identification of coat thickness as the main discriminating variable between seasons reinforces the importance of morphological traits as functional indicators of adaptation and resilience in small ruminants.

The increase in CT during winter is consistent with the insulating function of the coat in protecting against heat loss under colder conditions. Studies in small ruminants report seasonal increases in hair/fiber thickness and morphological changes that favor insulation and heat conservation during cold periods (Façanha et al. 2023; da Silva et al. 2025a, b). These responses have been documented in both sheep and goats and include morphological alterations of the coat layer and associated metabolic adjustments (Okourwa 2015; Ribeiro et al. 2016; Costa et al. 2018).

The observation of greater HL during summer, although initially appearing contrary to the classical expectation of longer hair for winter insulation, may be explained by species- or breed-specific factors, molting schedule, management practices, or differentiation between coat layers (e.g., undercoat versus guard hairs). In some populations, maximum hair length occurs at different phases (pre- or post-molt) and is strongly regulated by photoperiod and melatonin. Hormonal manipulations (e.g., melatonin) have been shown to clearly alter hair growth cycles in goats, demonstrating that seasonal hair length responses are complex and dependent on endogenous and exogenous factors (Sejian et al. 2018; Berihulay et al. 2019; Mascarenhas et al. 2023; Silveira et al. 2024). Thus, the observed pattern likely reflects the interaction between the natural molting cycle of the North Tinerfeña breed, local photoperiod, and possible management or selection effects.

Physiological responses to thermal load—such as increases in RT and RR during summer, particularly in the afternoon—are consistent with the literature on heat stress in goats, where exposure to high temperatures rapidly increases respiratory rate and, under more intense or prolonged conditions, elevates core body temperature. These are classic indicators of thermoregulatory effort through latent (evaporative) heat loss (panting) and limitations in convective and radiative heat dissipation (Gaughan et al. 2018; Maia et al. 2016). Such mechanisms have been described in both experimental heat stress protocols and field studies involving different goat breeds (Lima et al. 2022; Façanha et al. 2020; Ferreira et al. 2020).

The importance of CT as a discriminant variable between seasons—identified as the primary node in the CHAID decision tree—supports the hypothesis that coat morphology is one of the main determinants of thermal status and seasonal physiological and behavioral adjustment. In systems where CT varies markedly between seasons, this trait explains a substantial portion of interseasonal variability and can more directly predict insulation efficiency and the need for complementary physiological responses. Comparative studies among breeds have demonstrated that differences in CT account for a significant part of variation in heat gain and loss among genotypes (Silveira et al. 2024; da Silva et al. 2025a, b).

The discriminant analysis identifying CT and HL as the main variables separating winter and summer reinforces that thermal adaptations in this population are multicausal. While CT acts as a structural insulation variable, HL (and possibly fiber traits such as diameter and angle) modulates radiative and convective heat exchange. The combination of coat morphological traits with physiological responses (RT, RR, CST) and environmental variables (AT, RH, and RHL) provides an integrated representation of the adaptive profile, as also emphasized by reviews on thermal tolerance in goats, which highlight the importance of multiple anatomical, behavioral, and physiological mechanisms for maintaining homeothermy (Joy et al. 2020; Maldonado-Jáquez et al. 2025; Nair et al. 2021).

The main implications of this study lie in identifying phenotypic biomarkers, such as coat thickness, to characterize the seasonal thermal status of animals, which may be applied in management programs (e.g., decisions regarding shearing, and shelter provision) to reduce heat stress events. Additionally, because RR showed strong sensitivity to heat load and interaction with period of the day, routine monitoring of RR may serve as an early, low-cost indicator for the need for intervention (shade, water, and ventilation) during hot conditions.

These findings have broader implications in the context of climate change, where increasing temperatures and greater climatic variability are expected to challenge livestock systems worldwide. Understanding how locally adapted breeds modulate their thermoregulatory responses across seasons can support more effective conservation, breeding, and management strategies. Furthermore, the ability of North Tinerfeña goats to adjust their thermoregulatory mechanisms according to environmental conditions highlights the value of autochthonous genetic resources as potential reservoirs of adaptive traits that may contribute to the sustainability and resilience of future livestock production systems.

Although the physiological and morphological variables evaluated in this study provided valuable insights into the adaptive responses of goats to seasonal environmental variation, the inclusion of additional indicators could further improve the understanding of thermal adaptation mechanisms. Future research should integrate behavioral traits, such as feed and water intake, together with hematological, biochemical, and endocrine parameters, including cortisol concentrations, to provide a more comprehensive assessment of animal responses to environmental challenges. Such an integrative approach would contribute to a deeper understanding of the complex interactions between physiology, behavior, and metabolism underlying thermal resilience in goats raised under contrasting climatic conditions.

## Conclusion

North Tinerfeña goats exhibited distinct seasonal adaptive responses, combining morphological and physiological thermoregulatory mechanisms to maintain homeothermy under contrasting environmental conditions. During winter, coat traits favored heat conservation, whereas in summer, physiological adjustments played a greater role in heat dissipation. These findings highlight the adaptive capacity of this local breed and support the use of integrated physiological and morphological indicators for assessing thermal resilience in goats. 

## Electronic Supplementary Material

Below is the link to the electronic supplementary material.

Supplementary file 1 (DOCX 15.6 KB)

## Data Availability

The data that support the findings of this study are available from the corresponding author upon reasonable request.
