# Supplementary material for: Seasonal adaptive strategies of North Tinerfeña goats in the Canary Islands, Spain
Source: Int J Biometeorol. 2026 Oct 6;70(10):271. doi: 10.1007/s00484-026-03313-2 (PMC13642385; doi:10.1007/s00484-026-03313-2)
Supplement: Supplementary file 1 [file 484_2026_3313_MOESM1_ESM.docx]

| **Eigenvalue** | **Canonical correlation** | **Wilks’ λ** | **P-value** |
| --- | --- | --- | --- |
| 12.059 | 0.961 | 0.077 | <0.001 |
|  |  |  |  |
| **Variable** | **Standardized coefficient** | **Structure loading** | **Coefficient rank** |
| HL | 0.865 | 0.196 | 1 |
| CT | −0.850 | −0.503 | 2 |
| RT | −0.665 | −0.140 | 3 |
| RR | 0.636 | 0.266 | 4 |
| ST | 0.391 | 0.373 | 5 |
| ANGLE | 0.274 | −0.011 | 6 |
| HD | −0.131 | 0.010 | 7 |
|  |  |  |  |
| **Observed season** | **Predicted Winter** | **Predicted Summer** | **Correctly classified (%)** |
| Winter | 29 | 1 | 96.7 |
| Summer | 0 | 30 | 100.0 |
| **Overall** | **29** | **31** | **98.3** |

Table 4. Linear discriminant analysis of seasonal adaptive traits and grouped classification performance based on animal-season means
